# Horizontal Acquisition of a Multidrug-Resistance Module (R-type ASSuT) Is Responsible for the Monophasic Phenotype in a Widespread Clone of *Salmonella* Serovar 4,[5],12:i:-

**DOI:** 10.3389/fmicb.2016.00680

**Published:** 2016-05-10

**Authors:** Patricia García, Burkhard Malorny, M. Rosario Rodicio, Roger Stephan, Herbert Hächler, Beatriz Guerra, Claudia Lucarelli

**Affiliations:** ^1^Department of Functional Biology, Area of Microbiology, University of OviedoOviedo, Spain; ^2^Department of Biological Safety, Federal Institute for Risk AssessmentBerlin, Germany; ^3^Vetsuisse Faculty, National Centre for Enteropathogenic Bacteria and Listeria, Institute for Food Safety and Hygiene, University of ZurichZürich, Switzerland; ^4^Department of Infectious, Parasitic and Immuno-Mediated Diseases, Istituto Superiore di SanitàRome, Italy; ^5^European Public Health Microbiology Training Programme, European Centre for Disease Prevention and ControlStockholm, Sweden

**Keywords:** monophasic *Salmonella*, antimicrobial resistance, molecular epidemiology, European clone, plasmid origin

## Abstract

*Salmonella enterica* serovar 4,[5],12:i:- is a monophasic variant of *S*. Typhimurium incapable of expressing the second-phase flagellar antigen (*fljAB* operon), and it is recognized to be one of the most prevalent serovars causing human infections. A clonal lineage characterized by phage type DT193, PulseNet PFGE profile STYMXB.0131 and multidrug resistance to ampicillin, streptomycin, sulphonamides and tetracycline (R-type ASSuT) is commonly circulating in Europe. In this study we determined the deletions affecting the *fljAB* operon and the resistance region responsible for the R-type ASSuT in a strain of *Salmonella enterica* serovar 4,5,12:i:- DT193/STYMXB.0131, through an approach based on PCRs and Southern blot hybridization of genomic DNA. Using a set of nine specific PCRs, the prevalence of the resistance region was assessed in a collection of 144 *S. enterica* serovar 4,[5],12:i:-/ASSuT/STYMXB.0131 strains isolated from Germany, Switzerland and Italy. A 28 kb-region is embedded between the loci STM2759 and *iroB*, replacing the DNA located in between, including the *fljAB* operon. It encompasses the genes *bla*_TEM−1_, *strA-strB, sul2* and *tet*(B) responsible for the R-type ASSuT together with genes involved in plasmid replication and *orfs* of unknown function characteristically located on IncH1 plasmids. Its location and internal structure is fairly conserved in *S. enterica* serovar 4,[5],12:i:-/ASSuT/STYMXB.0131 strains regardless of the isolation source or country. Hence, in the *S. enterica* serovar 4,[5],12:i:-/ASSuT/STYMXB.0131 clonal lineage widespread in Germany, Switzerland and Italy, a resistance region derived from IncH1 plasmids has replaced the chromosomal region encoding the second flagellar phase and is an example of the stabilization of new plasmid-derived genetic material due to integration into the bacterial chromosome.

## Introduction

Multidrug resistant *Salmonella enterica* subsp. *enterica* serovar 4,[5],12:i:- (subsequently referred as *S*. 4,[5],12:i:-), a monophasic variant of *S*. Typhimurium, is recognized as an emerging public health hazard in Europe (EFSA, 2010, 2014; Hopkins et al., 2010; García et al., 2011, 2014; Gomes-Neves et al., 2014). It is mainly linked to the consumption of contaminated pork, and to a lesser extent, to other sources, such as poultry, cattle and companion animals (EFSA, 2010, 2014; García et al., 2011, 2014; Gomes-Neves et al., 2014). A clonal lineage of *S*. 4,[5],12:i:- characterized by phage type DT193, PulseNet pulsed field gel electrophoresis (PFGE) profile STYMXB.0131 (XBAI.0027 according to ECDC PFGE nomenclature; van Walle, 2013) and resistance to ampicillin, streptomycin, sulphonamides, and tetracycline (R-type ASSuT) is circulating in Europe (Hauser et al., 2010; Hopkins et al., 2010; Lucarelli et al., 2010; Gallati et al., 2013; Argüello et al., 2014; Barco et al., 2014). In this clonal lineage neither the resistance region nor the deletion affecting the *fljAB* operon has been determined. Instead in the Italian *S*. 4,5,12:i:- strain with PulseNet PFGE-XbaI profile STYMXB.0079 (XBAI.0096 according to ECDC) (Lucarelli et al., 2012) R-type ASSuT, the *fljAB* operon is affected by IS*26* insertion and the genes responsible for resistance are located in two genomic regions, named resistance region (RR) 1 and RR2 inserted into two adjacent loci of the bacterial chromosome. A similar region was recently detected in *S*. 4,5:i:-/ASSuT strains from Belgium (Boland et al., 2015). In contrast, *S*. 4,[5],12:i:-/ASSuT/STYMXB.0131 strains (mainly phage type DT193) does not harbor equivalent RR1 and RR2 regions.

Therefore, the aim of this study was to determine the deletion occurred at the *fljAB* locus and the genetic structure and location of the resistance region in a *S*. 4,5,12:i:-/ASSuT/DT193/STYMXB.0131 strain isolated in Germany. Furthermore, the occurrence of the resistance region was investigated within a collection of strains isolated over the period 2006 to 2012 in Germany, Switzerland and Italy from human, food and animal sources.

## Materials and methods

### Strains and genomic DNA extraction

A collection of 144 epidemiologically unrelated *S*. 4,[5],12:i:- strains with R-type ASSuT and PFGE-*XbaI* profile STYMXB.0131 isolated over the period 2006–2012 in Germany, Switzerland and Italy from human, food and animal sources were included in this study (Table S1). For PCR screening, DNA was extracted by thermal cell lysis from a 1 ml aliquot of an overnight culture grown in Luria-Bertani broth at 37°C. The strain 07-2006 (R-type ASSuT, phage type DT193, PFGE profile STYMXB.0131) isolated in 2007 from a pig lymph node in Lower Saxony (Germany) was selected to determine the deletion pattern affecting the *fljAB* operon and the chromosomal resistance region (Genbank accession no. KR856283). Genomic DNA of strain 07-2006 was purified using the DNeasy Blood & Tissue Kit (Qiagen, Hilden GmbH) according to the manufacturer's instruction.

### Characterization of deletions affecting the *fljAB* region

A set of 19 PCRs were performed to determine the genetic deletion responsible for the loss of the second-flagellar phase expression (*fljAB* and the surrounding genes) in the strain 07-2006. Usually a PCR contained 0.4 μM of each primer, 200 μM of each dNTP, 1.5 mM MgCl_2_, 10X PCR buffer, 1 U Platinum Taq polymerase (Invitrogen) and 10 ng of DNA template. PCR conditions were as follows: 95°C for 30 s, 33 cycles of 95°C for 30 s, 55–68°C for 30 s, 72°C for 1–3 min and a final extension at 72°C for 4 min. Primers-used, PCR cycling conditions and sizes of the expected fragments are shown in Table S2.

### Localization and genetic structure of the chromosomal resistance region

#### Long-range PCRs and DNA sequencing

Long-range PCRs were performed using QIAGEN LongRange PCR Kit (Qiagen GmbH, Hilden Germany). All PCRs were performed with Q-Solution. The final concentration of each primer in the reaction was 0.4 μM. As template 5–10 ng genomic DNA of strain 07-2006 was used. PCR conditions were as follows: 93°C for 3 min, 10 cycles of 93°C for 15 s, 62°C for 30 s and 68°C for 20 min followed by 28 cycles with increasing extension time for 20 s per each additional cycle. Primer-sets used in long-range PCR amplifications are shown in Table S3. Long-range PCR products were separated on a 0.8% agarose gel. The PCR fragment of the supposed size (main product) was cut from the agarose gel and purified with the illustra GFX PCR DNA and Gel Band Purification Kit (GE Healthcare Europe GmbH, München, Germany) according to the manufacturer's instruction. All PCR products were sequenced by Qiagen GmbH sequencing service (Hilden, Germany). For sequencing of long-range PCR products a primer walking strategy was applied. Oligonucleotide sequences used for sequencing can be obtained on request.

#### PCRs for linking long-range PCRs products

Gaps between adjacent long-range PCR products were completed by PCRs and consecutive DNA sequencing of the PCR products. Furthermore, genes STM2759 and *iroB* were completely sequenced. As indicated before, a PCR usually contained 0.4 μM of each primer, 200 μM of each dNTP, 1.5 mM MgCl_2_, 10X PCR buffer, 1 U Platinum Taq polymerase (Invitrogen) and 10 ng of DNA template. PCR conditions were as follows: 95°C for 30 s, 33 cycles of 95°C for 30 s, 55–56°C for 30 s, 72°C for 1–3 min and a final extension at 72°C for 4 min. Primers-used for PCRs and sequencing, PCR cycling conditions and size of resulting fragments are shown in Table S4.

#### Sequence assembling and analysis

Single DNA sequence runs obtained from both long-range and single PCRs were assembled to one contig and analyzed using Lasergene software package (version 8.1; DNASTAR, Madison, WI). Further genomic analysis and annotation was performed using Clone Manager 9 Professional (Scientific & Educational Software, Cary, NC). Sequence comparisons were performed using BLAST search at NCBI (http://blast.ncbi.nlm.nih.gov/).

#### Southern blot hybridization

The preparation of total DNAs of strain 07-2006 was performed using DNeasy Blood & Tissue Kit (Qiagen, Hilden GmbH). Total DNA (1 μg) was digested with FastDigest EcoRV (Fisher Scientific, Schwerte, Germany) and separated on a 1% agarose gel. As size standard DNA molecular weight marker MIII, DIG-labeled, (Roche Diagnostics GmbH, Mannheim, Germany) was used.

PFGE with XbaI enzyme was performed according to the PulseNet Europe protocol (Ribot et al., 2006) and using Lamda PFG Ladder (New England, Biolabs, Ipswich) as molecular weight marker.

Restriction fragments obtained by PFGE and total DNA digestion with EcoRV were transferred onto positively charged nylon membranes (Roche Diagnostics, GmbH, Mannheim, Germany) and hybridized with PCR-generated probes labeled with digoxigenin (DIG) for genes *bla*_TEM−1_, *sul2, tetA*(B), *merA*, STM2759, *iroB, gltS*, and *meth*Δ using primers listed in Table S5. Hybridizing bands were detected with the Detection Starter Kit (Roche Diagnostics GmbH).

### Prevalence of the identified chromosomal resistance region

**Figure 1:**
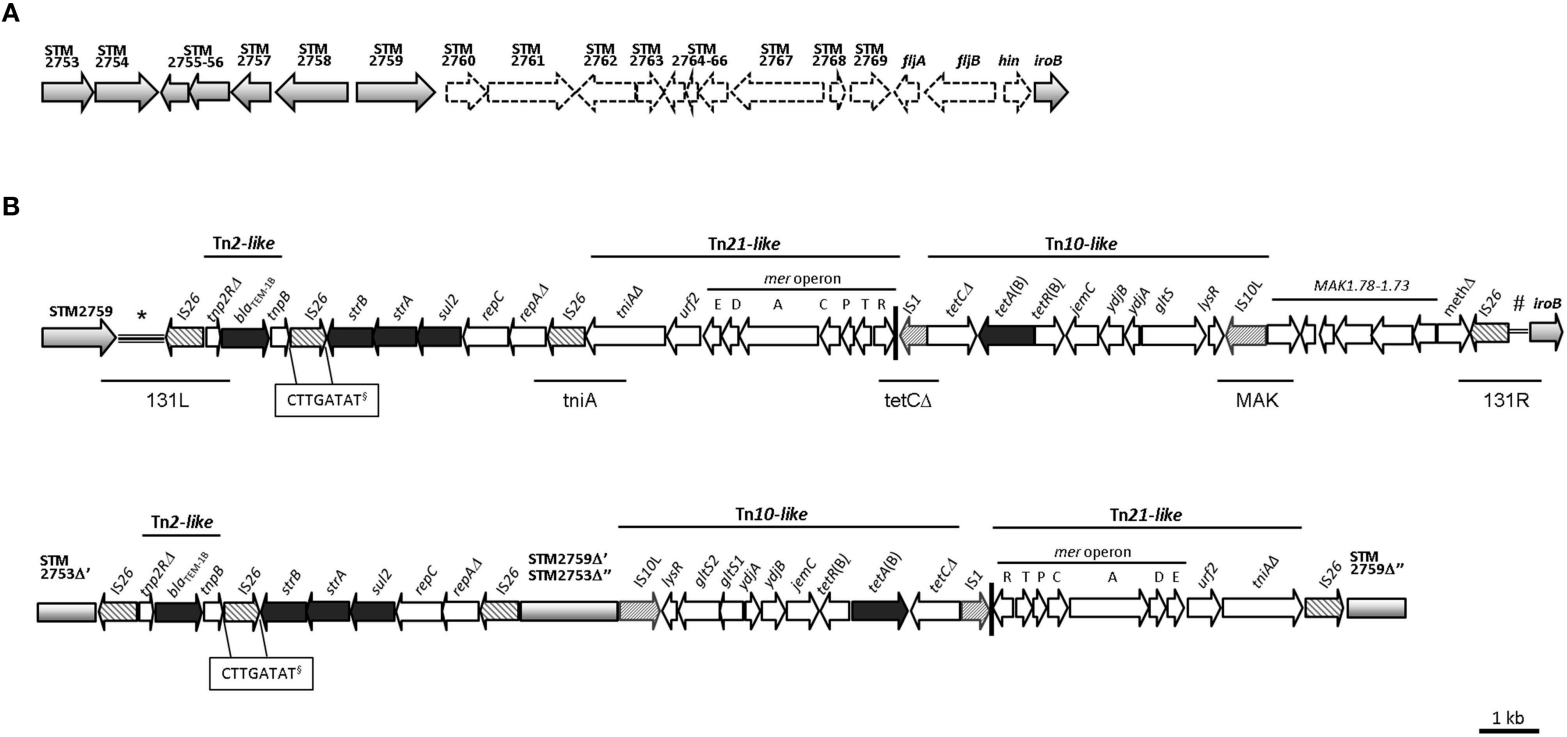
**(A)** Deletion pattern shown by the *S. enterica* subsp. *enterica* serovar 4,5,12:i:- strain 07-2006 in the chromosomal region encoding the second flagellar phase. The genetic map is based on *S*. Typhimurium LT2 (accession no. AE006468). The open reading frames (*orfs*) and the direction of their transcription are represented by gray (present) and white (absent) arrow*s*. **(B)** Comparison of the organization, genetic structure and location into the bacterial chromosome of the resistance regions of the strains 07-2006 (accession no. KR856283) and 105/7/03 (accession no. HQ331538). The open reading frames (*orfs*) and the direction of their transcription are represented by arrows: gray, chromosomal DNA; black, resistance genes; patterned, transposase genes of *IS* elements; white, other *orfs*. Vertical black bar indicates the 38 bp terminal imperfect IR of transposon Tn*21*. (^*^)indicates the intergenic region between the chromosomal loci STM2759 and STM2760. (#)indicates the intergenic region between the chromosomal genes *hin* and *iroB*. (§), DRs of IS*26*. Left and right junctions, and specific internal regions tested by PCR in a collection of strains are depicted underneath by horizontal lines.

The prevalence of the resistance region was assessed in 144 epidemiologically unrelated *S*. 4,[5],12:i:-/ASSuT/STYMXB.0131 strains performing specific PCRs for the resistance genes *bla*_TEM−1_, *strA*-*strB, sul2*, and *tet*(B) (Lucarelli et al., 2010), for the left (131L) and right (131R) junctions and for the internal region of the resistance region (*tniA, tetC*Δ, MAK) (Tables S1, S6; Figure 1B).

## Results

The resistance region (RR3) is a single complex module of 28,204 bp (Figure 1B) that comprises the genes *bla*_TEM−1_, *strA*-*strB, sul2* and *tet*(B) responsible for the R-type ASSuT, genes involved in plasmid replication and open reading frames (*orfs*) of unknown function. The module is inserted between two chromosomal loci, named STM2759 (encoding a putative phosphotransferase) and *iro*B (STM2773, a putative glycosyl transferase), causing the deletion of the genomic fragment located in between (15,716 bp). The latter comprises the genes STM2760–STM2769 (encoding different putative proteins), the *fljAB* operon (encoding a negative regulator of the *fliC* gene for the first-phase flagellar antigen and the second-phase flagellar antigen, respectively) as well as the *hin* gene (encoding the DNA invertase responsible for flagellar phase variation). The lacking of these genes in the genome of 07-2006 strain was confirmed by PCR amplification (Figure 1A).

In particular, the acquired DNA adjacent to STM2759 comprises a truncated Tn*2* transposon flanked by two copies of IS*26* in opposite orientation. This Tn*2*-like transposon consists of a partial resolvase gene (*tnp2R*Δ), the gene encoding a β-lactamase of type TEM-1B (*bla*_TEM−1_) and a presumptive transposase gene (*tnpB*). Downstream, the *strB* and *strA* phosphotransferase genes for streptomycin resistance, and *sul2* encoding a dihydropteroate synthase enzyme responsible for resistance to sulphonamides were found, followed by *repC* and *repA*Δ, plasmid replication genes, and a third copy of an IS*26* element.

Downstream a partial Tn*21* transposon included *tniA*Δ and *urf2* genes, encoding a putative transposase and a hypothetical protein, respectively, and the mercury resistance operon was found. After a defective Tn*10* transposon included an *IS*1, *tetC*(B)Δ gene, *tetR*(B), and *tetA*(B) genes encoding the tetracycline repressor and resistance proteins, respectively, *jemC, ydjB, ydjA, gltS*, and *lysR* genes, with hypothetical functions and a truncated IS*10*-left transposase. The Tn*10* transposon was followed by six *orfs* of unknown function together with a putative DNA modification methylase gene (*meth*Δ) truncated by another IS*26* element, which represent the right-hand end of the region.

In order to confirm the structure of the resistance region RR3, Southern blot hybridizations of genomic DNA were performed with relevant probes. According with the predicted structure of the nucleotide sequence (Genbank accession no. KR856283) two hybridization signals were obtained in the XbaI-pattern (Figure S1): a fragment of about 500 kb using probes for the genes *iroB, gltS* and *meth*Δ, and a fragment of estimated 30 kb for genes *bla*_TEM−1_, *sul2, tetA*(B), *merA*, and STM2759. The occurrence of a XbaI restriction site within *tetR*(B) (nt position 20,248) explains the separation of the observed hybridization signals. Likewise, a 13.6 and 4.9 kb fragments were observed in EcoRV-pattern (Figure S2) when using probes for *strA*-*strB* and *sul2*, or *bla*_TEM−1_ and STM2759, respectively. Altogether, these results confirmed the expected number of gene copies and position.

A PCR screening of 144 *S*. 4,[5],12:i:-/ASSuT/ STYMXB.0131 strains (Table S1) revealed that the resistance region is highly conserved in this clonal lineage along recent years (2006–2012) regardless of source (human, food, or animal) or country of isolation (Germany, Switzerland, or Italy; Table 1). In fact, 84% of strains (*n* = 121) were positive for all five PCRs, which include three internal targets and the left and right junctions of the resistance module. Thus, only 16% were negative for at least one of the five expected PCR products, being the more variable loci 131L (left junction) and *tniA* (internal junction between *repA*Δ and *tniA*Δ; Table S1).

**Table 1:** **Results of the PCRs for the presence of RR3 in 144 epidemiologically unrelated *S*. 4,[5],12:i:-/ASSuT/STYMXB.0131 strains**.

**PCR profile**					**Number of strains**			
**131L**	**tniA**	**tetCΔ**	**MAK**	**131R**	**Total (%)**	**Germany**	**Switzerland**	**Italy**
+	+	+	+	+	121 (84)	46	36	39
−	+	+	+	+	4 (2.8)	0	0	4^*^
−	−	+	+	+	2 (1.4)	1	0	1
−	+	−	-	+	1 (0.7)	1	0	0
−	+	+	+	−	2 (1.4)	1	0	1
−	−	−	+	−	1 (0.7)	0	0	1
+	−	+	+	+	8 (5.6)	3	4	1
+	+	+	−	+	1 (0.7)	1	0	0
+	+	+	+	−	4 (2.8)	1	2	1
TOTAL					144 (100)	54	42	48

*PCR-profile is based on the amplicons represented in Figure 1. Individual features of strains are compiled in Table S1*.

* *indicates a fragment with aberrant molecular weight*.

## Discussion

The resistance region (RR3) is a single complex module that comprises the genes responsible for the R-type ASSuT, genes involved in plasmid replication and open reading frames (*orfs*) of unknown function. The module is inserted between two chromosomal loci, named STM2759 and *iro*B (STM2773), causing the deletion of the genomic fragment located in between. Regarding the flanking chromosomal DNA of RR3, it shows high-level sequence identity to the corresponding regions of the biphasic *S*. Typhimurium LT2 genome (GenBank accession no. AE006468). Indeed, 99% sequence identity was observed for the segment located upstream of the resistance module, spanning from the 5′ end of STM2758 to the intergenic region between STM2759 and STM2760, and 100% for the downstream segment, that includes the *iroB* gene and the intergenic non-coding sequence next to *hin*. Previous studies have described different deletion patterns affecting the *fljAB* operon and surrounding genes as a consequence of a single IS*26* insertion (Soyer et al., 2009; EFSA, 2010; Laorden et al., 2010; Lucarelli et al., 2012; García et al., 2013; Boland et al., 2015). In contrast, a large resistance region bounded by direct copies of IS*26* along with a 15-kb deletion was found at this location in the *S*. 4,[5],12:i:-/ASSuT/STYMXB.0131 strain, being responsible for the monophasic phenotype. However, the IS*26* located at the *fljAB* operon is inserted at the same nt position (2,916,036 of GenBank accession no AE006468) of the intergenic region between the *hin* and *iroB* genes as in the 4 strains described by Lucarelli et al. (2012) and in the chromosomal resistance regions identified in *S*. 4,5:i:- strains from Belgium (GenBank accession no. KJ999732; Boland et al., 2015). According to previous observations (Lucarelli et al., 2012; García et al., 2013; Boland et al., 2015), our results support the hypothesis that the region encoding the second-phase flagellar antigen, having a lower average of GC content in comparison with the *Salmonella* core genome (45 vs. 52.2%), could be an integration hotspot for foreign DNA (Bäumler and Heffron, 1998). Therefore, it could be hypothesized that one copy of IS*26* located at the *fljAB* region could be acted as a target or recognition site for the incorporation of additional antibiotic resistance gene(s) by another IS*26* element, and so on (Reid et al., 2015). These IS*26*-mediated events may occur sequentially explaining the acquisition of multiple tandem copies of IS*26* (four in total) and leading the assembly of the final resistance region identified in the chromosome of the *S*. 4,[5],12:i:-/ASSuT/STYMXB.0131 strain.

The region including the three copies of IS*26* elements (Figure 1) shows 99% sequence identity with RR1, also named as Tn*6029E* in Reid et al. (2015), located in the chromosome of the Italian *S*. 4,5,12:i:- ASSuT/STYMXB.0079 strain (GenBank accession no. HQ331538) and 100% sequence identity with the Belgium *S*. 4,5:i:-/ASSuT strain (GenBank accession no. KJ999732). As reported by others (Hall and Cain, 2012), this element could correspond to an intermediate structure in the derivation of transposon Tn*6029* which differs only in the reversed orientation of the segment *strA*-*strB*-*sul2*-*repC*-*repA*Δ (Cain and Hall, 2012), or be related with part of the transposon Tn*6026* after the inversion of the same genetic segment (Reid et al., 2015). In fact, a BLAST search (NCBI, February 2016) revealed that the region identified in this study shares high-level identity with these composite transposons identified in other bacterial chromosomes such as *E. coli* O104:H4 (GenBank accession no. CP003297 and CP003301; Ahmed et al., 2012; Chowdhurry et al., 2015) or *S*. Typhi (SGI1, GenBank accession no. KM023773; Chiou et al., 2014); as well as carried by a number of IncH1 plasmids, for example pO111_1 (*E. coli*, GenBank accession no. AP010961; Ogura et al., 2009), pSRC27-H (*S*. Typhimurium, GenBank accession no. HQ840942; Cain and Hall, 2012) and p109/9 (*S*. Typhimurium, GenBank accession no. KP899805).

The composite structure of incompletes Tn*21* and Tn*10* transposons shows a 99% of nucleotide identity with the RR2 region of the *S*. 4,5,12:i:- ASSuT/STYMXB.0079 Italian strain but with some differences: (i) the segment stands inverted with Tn*21*-like at the left side; (ii) the *gltS* gene encodes a single hypothetical protein lacking the frameshift detected by Lucarelli et al. (2012); and (iii) IS*10*L is not contiguous with *S*. Typhimurium chromosomal DNA. Indeed, immediately downstream of the Tn*10*-like transposon and adjacent to *iroB* a segment of DNA, not present in RR2, was identified. It comprises six *orfs* of unknown function together with a putative DNA modification methylase gene (*meth*Δ) truncated by another IS*26* element. BLAST analysis (NCBI, February 2016) of these additional *orfs* (4709 bp; nt 25,595–30,303) revealed they are commonly part of the conserved backbone of IncH1 plasmids showing 99% sequence identity with nine of them: pO111_1 (*E. coli*, GenBank accession no. AP010961; Ogura et al., 2009), pSRC27-H (*S*. Typhimurium, GenBank accession no. HQ840942; Cain and Hall, 2012), p109/9 (*S*. Typhimurium, GenBank accession no. KP899805), pB71 (*S*. Typhimurium, GenBank accession no. KP899806), pF8475 (*S*. Typhimurium, GenBank accession no. KP899804), pMAK1 (GenBank *S*. Choleraesuis, accession no. AB366440), pHCM1 (*S*. Typhi, GenBank accession no. AL513383; Parkhill et al., 2001), as well as pEQ1 and pEQ2, two sequenced plasmids recovered from equine *E. coli* strains (GenBank accession no. KF362121 and KF362122; Dolejska et al., 2014). It is remarkable that both Tn*21* and Tn*10*, either truncated or entire, and contiguous or not to Tn*6029*, have been detected among IncH1 plasmids. Therefore, it could be hypothesized that in strain *S*. 4,5,12:i:-/STYMXB.0079 and in strain *S*. 4,5,12:i:-/STYMXB.0131 two independent and different acquisitions of the same regions from IncH1 plasmids occurred, with exception of the six *orfs* present only in the latter strain. Similar events of DNA acquisition have been proposed for the *S*. 4,5:i:- Belgium strain which harbors part of the Tn*21*-Tn*10*-like structure and the mentioned IncH1 region identified in *S*. 4,5,12:i:-/STYMXB.0131 (Boland et al., 2015).

Despite the presence of numerous transposable elements (four copies of IS*26*, one copy of IS*1* and one copy of IS*10*), a PCR screening of 144 *S*. 4,[5],12:i:-/ASSuT/ STYMXB.0131 strains revealed that the resistance region is highly conserved in 84% of strains regardless of source or country of isolation. The observed variability in 16% of strains could be explained, at least in part, by the repeated presence of IS*26* which could recombine yielding different genetic variants. In addition, the presence of the IS*26* in the same position of the *fljAB* operon also in other strains (Lucarelli et al., 2012; Boland et al., 2015) suggest the existence of a common ancestor harboring an IS*26* in this position, where occurred IS*26*-mediated homologous recombination events, responsible for (i) acquisition of the new region present in the chromosome of *S*. 4,[5],12:i:-/ASSuT/ STYMXB.0131 07-2006, and (ii) deletion of the second phase flagellar antigen genes.

In conclusion, this study describes a resistance region identified on the chromosome of a clonal lineage of monophasic *S*. 4,[5],12:i:- widely distributed in at least Germany, Switzerland, and Italy. The region has replaced genes responsible for the expression of the second phase flagellar antigen in biphasic *S*. Typhimurium strains and represents an example of the stabilization of new plasmid-derived material into the bacterial chromosome.

Further studies are needed in order to establish if mercury operon is expressed and if this resistance island could have any effect on the bacterial physiology, as well as metabolic pathways, growth, and virulence.

## Author contributions

PG, BM, and MR were responsible for sequencing the structure of the genomic resistance region. BM, RS, HH, CL provided further strains and characterized them. PG, BM, BG, and CL drafted the manuscript. PG, BM, HH, and CL designed the study and edited the manuscript. All authors read, commented on, and approved the final manuscript.

## Funding

This work was supported by the Federal Institute for Risk Assessment (BfR: project no.s 46-001 and 46-003, Germany), the “Fondo de Investigación Sanitaria” of the “Instituto de Salud Carlos III” (FIS PI11-00808, Spain), co-funded by European Regional Development Fund of the European Union: a way to making Europe. PG was the recipient of a grant from the “Fundación para el Fomento en Asturias de la Investigación Científica Aplicada y la Tecnología” (FICYT, Ref. BP08-031). She performed a short stay at the Department of Biological Safety of the Federal Institute for Risk Assessment (BfR), Berlin, Germany, supported by the same grant. This work was partly supported by a grant from the Italian Ministry of Health Centro Nazionale per la Prevenzione ed il Controllo delle Malattie (CCM; project “Sorveglianza delle malattie trasmesse da alimenti e acqua (EnterNet): adeguamento del sistema italiano al quadro normativo europeo”) and by the Swiss Federal Office of Public Health, Division Communicable Diseases.

### Conflict of interest statement

The authors declare that the research was conducted in the absence of any commercial or financial relationships that could be construed as a potential conflict of interest.
