# Supplementary material for: Horizontal Acquisition of a Multidrug-Resistance Module (R-type ASSuT) Is Responsible for the Monophasic Phenotype in a Widespread Clone of Salmonella Serovar 4,[5],12:i:-
Source: Front Microbiol. 2016 May 10;7:680. doi: 10.3389/fmicb.2016.00680 (PMC4861720; doi:10.3389/fmicb.2016.00680)
Supplement: Supplementary file 1 [file Presentation1.pdf]

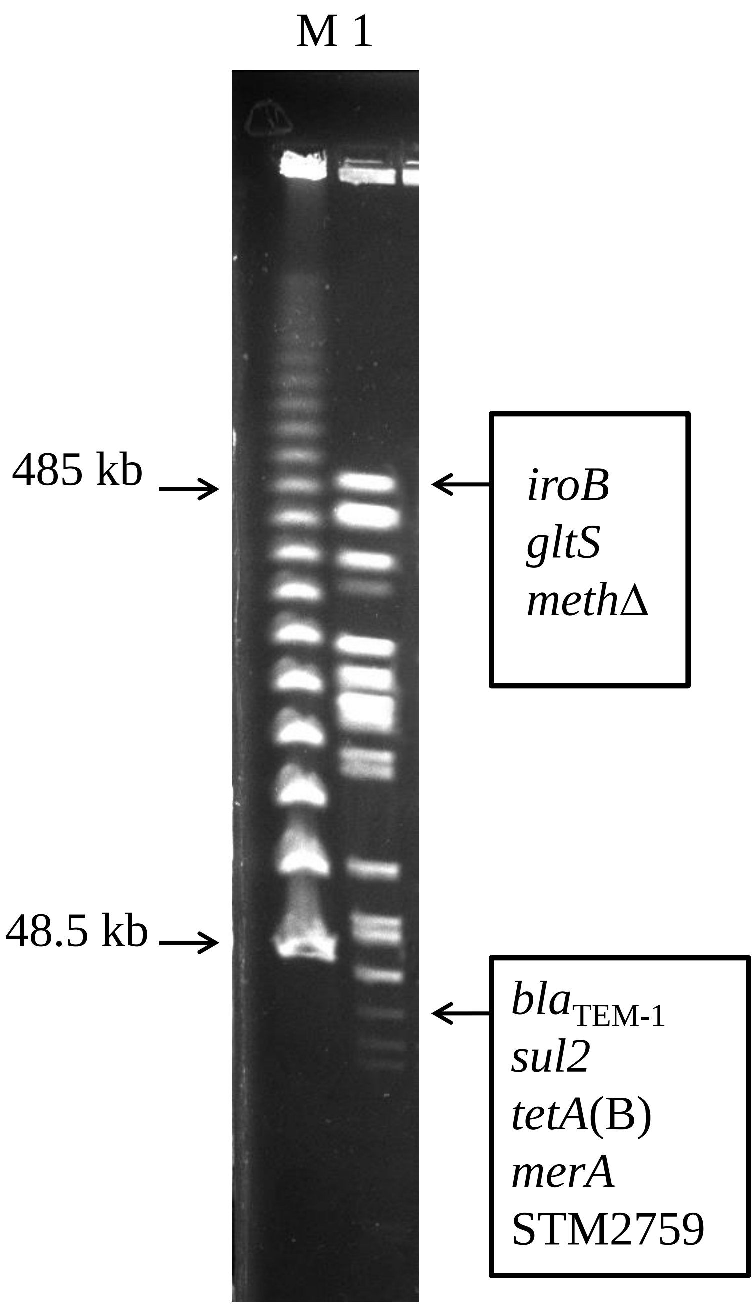

Supplementary Figure S1.
Pulsed field-gel electrophoresis with XbaI on strain 07-2006 (lane 1). Lane M is the molecular weight marker (Lambda PFG ladder). Arrows on the left indicate which genes resulted located on the two fragments by southern blot hybridization.
