# Supplementary material for: Horizontal Acquisition of a Multidrug-Resistance Module (R-type ASSuT) Is Responsible for the Monophasic Phenotype in a Widespread Clone of Salmonella Serovar 4,[5],12:i:-
Source: Front Microbiol. 2016 May 10;7:680. doi: 10.3389/fmicb.2016.00680 (PMC4861720; doi:10.3389/fmicb.2016.00680)
Supplement: Supplementary file 2 [file Presentation2.pdf]

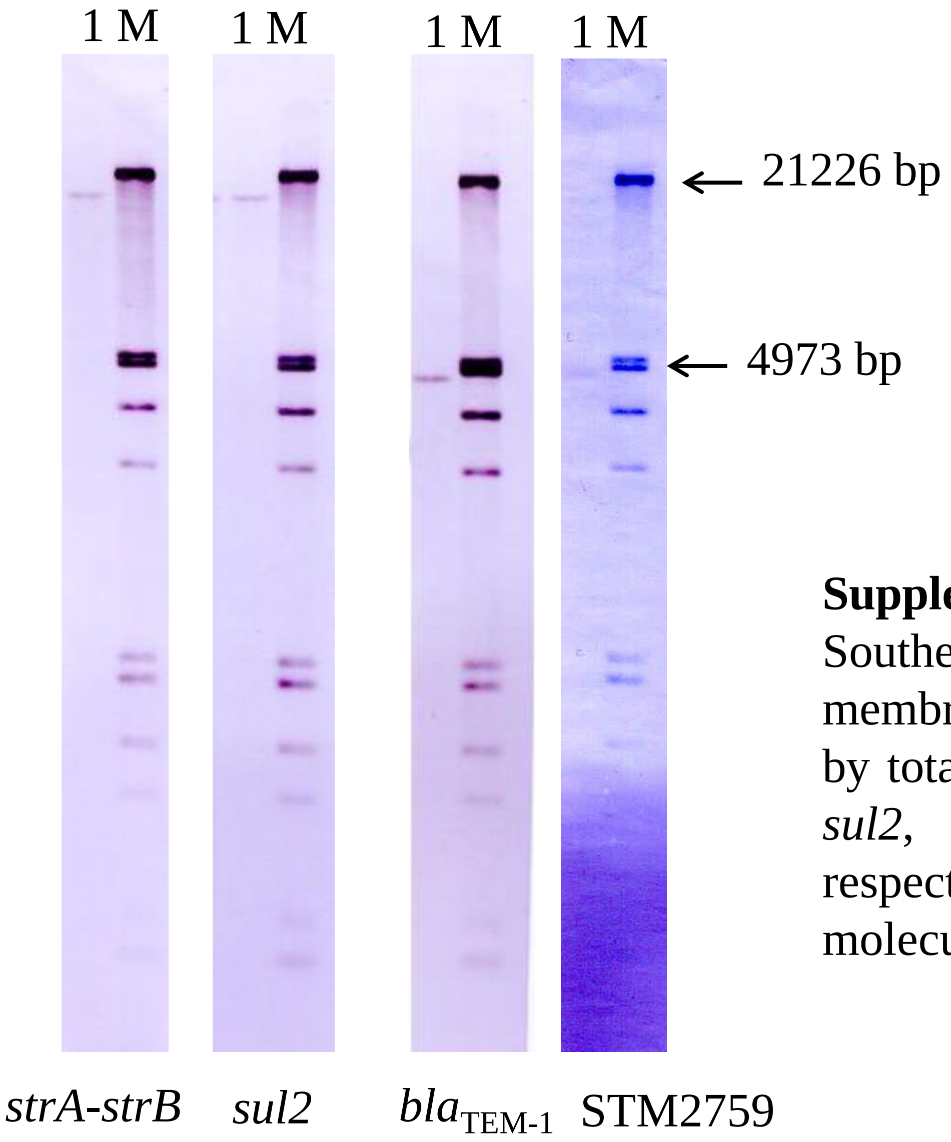

Supplementary Figure S2.
Southern blot hybridization was performed on a membrane containing restriction fragments obtained by total DNA digestion with EcoRV, with *strA*-*strB*, *sul2*, *bla*$_{\text{TEM-1}}$, and STM2759 gene probes, respectively. Lane 1: strain 07-2006. Lane M is the molecular weight marker lane (Marker MIII, Roche).
