# Supplementary material for: Horizontal Acquisition of a Multidrug-Resistance Module (R-type ASSuT) Is Responsible for the Monophasic Phenotype in a Widespread Clone of Salmonella Serovar 4,[5],12:i:-
Source: Front Microbiol. 2016 May 10;7:680. doi: 10.3389/fmicb.2016.00680 (PMC4861720; doi:10.3389/fmicb.2016.00680)
Supplement: Supplementary file 4 [file Table2.DOC]

**Table S2**. Primer-sets used in PCRs for determining deletions affecting the *fljAB* region. Primers designed based on *S.* Typhimurium LT2 genome, accession number AE006468.

| **Name** | **Sequence (5’ to 3’)** | **Target** | **Amplicon size (bp)** | **Annealing** | **Elongation** |
| --- | --- | --- | --- | --- | --- |
| stm2753-f1 | TGCAGAGCCTGCTGACAATG | STM2753 | 455 | 56°C, 30 s | 72°C, 1 min |
| stm2753-r1 | AGCCTGACGAGTGAATAGAC | STM2753 |
| stm2753-f2 | CGGCAAACACCATATGAACG | STM2753 | 1054 | 56°C, 30 s | 72°C, 1 min |
| stm2753-r1 | AGCCTGACGAGTGAATAGAC | STM2753 |
| stm2754-f1 | AAGTGCACGCGGCCAATTTC | STM2754 | 971 | 56°C, 30 s | 72°C, 1 min |
| stm2754-r1 | GCTCTCCATCTTTCCCATAC | STM2754 |
| stm2758-f1 | GTTTCAGCAGCGCTTTGTCC | STM2758 | 902 | 56°C, 30 s | 72°C, 1 min |
| stm2758-r1 | AGCGATTTGTGCCGATAGTG | STM2758 |
| stm2759-f1 | CTGTTCGGTGCGTAATTGTC | STM2759 | 298 | 56°C, 30 s | 72°C, 1 min |
| stm2759-r1 | GCGGATCCAGATTGCGATA | STM2759 |
| stm2759-f1 | CTGTTCGGTGCGTAATTGTC | STM2759 | 769 | 56°C, 30 s | 72°C, 1 min |
| stm2759-r2 | CCTGATGCCAGTGTTGATTG | STM2759 |
| stm2759-f2 | CAATCAACACTGGCATCAGG | STM2759 | 1560 | 56°C, 30 s | 72°C, 2 min |
| stm2759-r3 | GTCTAAAGAGGCGGTACCAA | STM2759 |
| stm2760-f2 | TTAGACGGAGCGTATCTCTG | STM2760 | 674 | 56°C, 30 s | 72°C, 1 min |
| stm2760-r2 | TTCTGACTGGCTGAGTTGTG | STM2760 |
| stm2760-f1 | TGGACCGGGTTCATCATATC | STM2760 | 407 | 56°C, 30 s | 72°C, 1 min |
| stm2760-r1 | GACCCTGCTCTTCGTAATAG | STM2760 |
| stm2761-f1 | TCCGGGACCAACGTTTACAG | STM2761 | 1348 | 56°C, 30 s | 72°C, 2 min |
| stm2761-r1 | CAGGTGTCAGATGGGCATAC | STM2761 |
| stm2763-f1 | CCTGAATGAGACCCACTAAG | STM2763 | 654 | 56°C, 30 s | 72°C, 1 min |
| stm2763-r1 | CGTTGGCCCAGATTATCTGC | STM2763 |
| stm2766-r1 | TCCAGAGCGGACTAAGTAC | STM2766 | 1581 | 56°C, 30 s | 72°C, 2 min |
| stm2767-f2 | GAGCCTGAATGGGATTCTTG | STM2767 |
| stm2767-r1 | TTCCAAAGCCACTCTGGAAG | STM2767 | 1098 | 56°C, 30 s | 72°C, 2 min |
| stm2767-f1 | TCGCGGCCTTCTGGAGAATG | STM2767 |
| stm2769-f1 | TCGCGGCCTTCTGGAGAATG | STM2768 | 986 | 56°C, 30 s | 72°C, 1 min |
| stm2769-r1 | AACTGGCCATTCTCCAGAAG | STM2768 |
| ST-fljA-for | GTATGGCTGTAAATGATATTTCC | *fljA* | 527 | 58°C, 30 s | 72°C, 1 min |
| ST-fljA-rev | CAGCGTAGTCCGAAGACGTGA | *fljA* |
| Fsa2 | CAAGTAATCAACACTAACAGTC | *fljB* | 1514 | 56°C, 30 s | 72°C, 2 min |
| rFsa2 | TTAACGTAACAGAGACAGCAC | *fljB* |
| fljB-s | ACGAATGGTACGGCTTCTGTAACC | *fljB* | 526 | 68°C, 30 s | 72°C, 1 min |
| fljB-as | TACCGTCGATAGTAACGACTTCGG | *fljB* |
| ST-HIN-L | TTGGGTATATTCGGGTGT | *hin* | 545 | 48°C, 30 s | 72°C, 1 min |
| ST-HIN-R | TATACTGCTTGCCGGAAA | *hin* |
| iroB-F | TGCGTATTCTGTTTGTCGGTCC | *iroB* | 605 | 60°C, 30 s | 72°C, 1 min |
| iroB-R | TACGTTCCCACCATTCTTCCC | *iroB* |
