# Supplementary material for: Horizontal Acquisition of a Multidrug-Resistance Module (R-type ASSuT) Is Responsible for the Monophasic Phenotype in a Widespread Clone of Salmonella Serovar 4,[5],12:i:-
Source: Front Microbiol. 2016 May 10;7:680. doi: 10.3389/fmicb.2016.00680 (PMC4861720; doi:10.3389/fmicb.2016.00680)
Supplement: Supplementary file 5 [file Table3.DOC]

**Table S3.** Primer-sets used in long-range PCRs*.* Primers designed based on *S.* 4,5,12:i:- 105/7/03 genome, accession number HQ331538.

| **Name** | **Sequence (5’ to 3’)** | **Target** | **Amplicon size (bp)** |
| --- | --- | --- | --- |
| R-iroB-R | CGCCAGAGACAGCACAGGGTATAG | *iroB* | 7812 |
| LR-gltS-F | TTGGTATGGGAGCAACACCAACAG | *gltS* |
| LR-gltS-r6 | AGAATTAATTCGTAAACGTGCAAGCTA | *gltS* | 3934 |
| LR-tetB-R(#1369) | CGTTGGCAAGACTGGCATGATAAGG | *tet*(B) |
| LR-tetB-F | GCAACCGGTGTTATTGGCCCATTAC | *tet*(B) | 4099 |
| LR_merA-2r | ACTTTCTCCAGGGCGTCCTTGACATGCA | *merA* |
| LR_merA-1f | GCGCAGACCTTCAACAAGGATGTGA | *merA* | 5654 |
| LR-sul2r1 | GCTTGCGTCGCGGGTTGATAACTG | *sul2* |
| LR-sul2f-1 | TTCTCCGATGGAGGCCGGTATCTG | *sul2* | 3941 |
| LR-TEM-f1 | GATGGTAAGCCCTCCCGTATCGTAG | *bla*_TEM-1_ |
| LR-strB-f | CTTATGGGTGCCTTTCCGCAGCTTG | *strB* | 4085 |
| LR-stm2759-f1 | TGGCTGCTATGTCAGGTTGGTCAAG | STM2759 |
