# Supplementary material for: Horizontal Acquisition of a Multidrug-Resistance Module (R-type ASSuT) Is Responsible for the Monophasic Phenotype in a Widespread Clone of Salmonella Serovar 4,[5],12:i:-
Source: Front Microbiol. 2016 May 10;7:680. doi: 10.3389/fmicb.2016.00680 (PMC4861720; doi:10.3389/fmicb.2016.00680)
Supplement: Supplementary file 6 [file Table4.DOC]

**Table S4.** Primer-sets used in PCRs for gap closure of long-range PCRs.

| **Name** | **Sequence (5’ to 3’)** | **Target** | **Amplicon size (bp)** | **Annealing** | **Elongation** |
| --- | --- | --- | --- | --- | --- |
| tetR-r1 | GACCTCATTAAGCAGCTCTA | *tetR* | 1407 | 55°C, 30 s | 72°C, 1 min |
| dtetC-r1 | TTGGGTTATCAAGAGGGTCA | *tetC* |
| merC-f1 | GGGTGTCGATCTGGGACTTC | *merA* | 1987 | 56°C, 30 s | 56°C, 2 min |
| merD-r1 | CAAGGCCGCATCGTCGAACA | *merA* |
| Tnp3R-f1 | GACGTCAGGTGGCACTTTTC | *tnp2R*Δ | 1300 | 56°C, 30 s | 72°C, 2 min |
| tnpB-r1 | AACCGCGACGCTTACAGCT | *tnpB* |
| tnpIS26-3r | CTTTGAATGGGTTCATGTGCA | IS*26* | 1663 | 55°C, 30 s | 72°C, 3 min |
| iroC-r2 | GCGAACTATCCAGGCACGA | *iroC* |
| stm2759-r3 | GTCTAAAGAGGCGGTACCAA | STM2759 | 2671 | 55°C, 30 s | 72°C, 3 min |
| STM2758-r2 | CGATGCTTTGTAACATTTGCA | STM2758 |
