# Supplementary material for: Horizontal Acquisition of a Multidrug-Resistance Module (R-type ASSuT) Is Responsible for the Monophasic Phenotype in a Widespread Clone of Salmonella Serovar 4,[5],12:i:-
Source: Front Microbiol. 2016 May 10;7:680. doi: 10.3389/fmicb.2016.00680 (PMC4861720; doi:10.3389/fmicb.2016.00680)
Supplement: Supplementary file 7 [file Table5.DOC]

**Table S5.** Primer-sets used to generate DIG-labelled probes for hybridization.

| **Name** | **Sequence (5’ to 3’)** | **Target** | **Amplicon size (bp)** |
| --- | --- | --- | --- |
| STM2759-f1 | CTGTTCGGTGCGTAATTGTC | STM2759 | 770 |
| STM2759-r2 | CCTGATGCCAGTGTTGATTG | STM2759 |
| STM2759-f2 | CAATCAACACTGGCATCAGG | STM2759 | 1561 |
| STM2759-r3 | GTCTAAAGAGGCGGTACCAA | Intergenic STM2759-RR3 |
| TEM-1 | TTGGGTGCACGAGTGGGT | *bla*_TEM-1_ | 504 |
| TEM-2 | TAATTGTTGCCGGGAAGC | *bla*_TEM-1_ |
| strB-2 | GGATCGTAGAACATATTGGC | *strB* | 1188 |
| strA-1 | TGGTGATAACGGCAATTC | *strA* |
| Sul2-1 | TCAACATAACCTCGGACAGT | *sul2* | 707 |
| Sul2-2 | GATGAAGTCAGCTCCACCT | *sul2* |
| merA-1 | ACCATCGGCGGCACCTGCGT | *merA* | 1238 |
| merA-2 | ACCATCGTCAGGTAGGGGAACAA | *merA* |
| tetB-1 | TTGGTTAGGGGCAAGTTTTG | *tet*(B) | 659 |
| tetB-2 | GTAATGGGCCAATAACACCG | *tet*(B) |
| gltS-f2 | TCCTCTATCGGGCTAAGTTC | *gltS* | 829 |
| gltS-r3 | GTGTTGCTCCCATACCAAAG | *gltS* |
| dmeth-2f | TGGTGCAGGTTGCAAGTATC | *dmeth* | 545 |
| dmeth-1r | CTGGGTGTCGCATTCAAAGC | *dmeth* |
| iroB-F | TGCGTATTCTGTTTGTCGGTCC | *iroB* | 606 |
| iroB-R | TACGTTCCCACCATTCTTCCC | *iroB* |
