# Supplementary material for: Horizontal Acquisition of a Multidrug-Resistance Module (R-type ASSuT) Is Responsible for the Monophasic Phenotype in a Widespread Clone of Salmonella Serovar 4,[5],12:i:-
Source: Front Microbiol. 2016 May 10;7:680. doi: 10.3389/fmicb.2016.00680 (PMC4861720; doi:10.3389/fmicb.2016.00680)
Supplement: Supplementary file 8 [file Table6.DOC]

**Table S6.** Primer-sets used for PCR screening of the resistance region RR3 in other *S.* 4,[5],12:i:-/ASSuT/STYMXB.0131 strains*.*

| **Target region** | **Primer** | **Sequence (5’ to 3’)** | **Amplicon size (bp)** | **Position in KR856283** |
| --- | --- | --- | --- | --- |
| 131L | STM2759 | GTTTACACGACTGCCTGAA | 2819 | 1681-1699 |
|  | TEM(2) | GGATAATACCGCACCACATA |  | 4481-4500 |
| *tniA* | repAΔ(2) | GCAGGTAGATCACCGGGC | 2008 | 10762-10779 |
|  | tniAΔ(2) | CTCGGCGCCAGGTATACG |  | 12753-12770 |
| *tetC*Δ | merR^a^ | AACCTGACCATTGGCGTTTTT | 1337 | 17258-17278 |
|  | tetCΔ^a^ | GGCATCACTTCTTGGATAGG |  | 18576-18595 |
| MAK | lysR(2) | ACCCGCGGCGAGTAAAGC | 1918 | 24205-24222 |
|  | MAK1.78 | GTCCTTCAAAATCTATATCAGG |  | 26102-26123 |
| 131R | methΔ | ACTTATTCGCCAGATCAAAGG | 1836 | 29764-29784 |
|  | iroB | CTTCGGCTGCTTTCTGTGC |  | 31582-31600 |
